# Genetic diversity and haplotype distribution patterns analysis of cytb and RAG2 sequences in *Rana hanluica* from southern China

**DOI:** 10.3389/fgene.2024.1374263

**Published:** 2024-05-20

**Authors:** Zeshuai Deng, Yuan Li, Zhiwei Gao, Zhiqiang Zhang, Daode Yang

**Affiliations:** Institute of Wildlife Conservation, Central South University of Forestry and Technology, Changsha, China

**Keywords:** *Rana*, genetic diversity, haplotype diversity, species dispersal, phylogenetic relationships

## Abstract

*Rana hanluica*: an endemic amphibian of China, is found in the hills and mountains south of the Yangtze River. In this comprehensive study, we collected 162 samples from 14 different localities to delve into the genetic diversity of *Rana hanluica* using mitochondrial Cytb and nuclear RAG2 as genetic markers. Our findings reveal that the Nanling Mountains, specifically regions like Jiuyi Shan, Jinggang Shan, Mang Shan, and Qiyun Shan, are genetic hotspots harboring remarkable diversity. The research results also indicate that there is gene flow among the various populations of the species, and no distinct population structure has formed, which may be due to migration. Moreover, populations in some regions, as well as the overall population, show signs of a possible genetic bottleneck, which we speculate may have been caused by climate change. However, given the exploratory nature of our study, further investigations are warranted to confirm these observations. Through phylogenetic analyses, we uncovered indications that *R. hanluica* might have originated within the Nanling region, dispersing along the east-west mountain ranges, with a significant contribution originating from Jiuyi Shan. The genetic distributions uncovered through our research reflect historical migratory patterns, evident in the distinct haplotypes of the RAG2 gene between the western and eastern parts of the studied area. Moreover, Heng Shan and Yangming Shan exhibited unique genetic signatures, possibly influenced by geographic isolation, which has shaped their distinct genotypes. The insights gained from this study hold profound implications for conservation efforts. By identifying regions rich in genetic diversity and crucial gene flow corridors, we can develop more effective conservation strategies. Preserving these genetically diverse areas, especially within the Nanling Mountains, is vital for maintaining the evolutionary potential of *R. hanluica*. In conclusion, our research has laid a solid foundation for understanding the genetic landscape of *R*. *hanluica*, shedding light on its origins, population structures, and evolutionary trajectories. This knowledge will undoubtedly guide future research endeavors and inform conservation strategies for this endemic amphibian.

## Introduction

Southern China lies within the second-level terrain (Terrain II) and the third-level terrain (Terrain III), extending from the eastern part of the Qinghai-Tibet Plateau to the Pacific Ocean. Its diverse ecosystem, shaped by numerous mountains, rivers, and lakes, endows the area with high levels of biodiversity and endemism (Myers, et al., 2000; Qian and Ricklefs, 2000). The heterogeneity in topography and climate provides ample habitats, fostering species diversity (Myers et al., 2000). The complex terrain of south China likely contributes to shaping patterns of species diversification in the region. Especially, the Lingnan region, including the Nanling Mountains, is one of the hotspots rich in species diversity in China (Ministry of Ecology and Environment of the People’s Republic of China https:// www.mee.gov.cn/). Over the years, many new amphibian species have been reported in and around this area (Li et al., 2020; Lyu et al., 2020). However, our understanding of the processes governing species diversification and distribution in Southern China remains limited and necessitates further research.

Investigating genetic diversity and determining the spatial distribution of amphibian species are of paramount significance for their conservation. The genetic variation in the genus *Rana* has received extensive attention (Kim et al., 1999; Zhan et al., 2009; Zhou et al., 2012; Chen et al., 2022). Currently, habitat degradation or loss, illegal capture, and environmental pollution are the most serious threats to amphibians in China (Jiang et al., 2016). *Rana hanluica,* a native Chinese species, was first characterized in 2007. and belongs to the Anura order, Ranidae family, and *Rana* genus. Despite its extensive geographic range and classification as Least Concern (LC) in the Chinese Red List due to its broad distribution, it maintains a Data Deficient (DD) status on the IUCN Red List. Initially observed in Hunan, subsequent sightings have expanded its territory to Jiangxi, Zhejiang, Guizhou, and Chongqing (Shen et al., 2007; Jiang et al., 2021). *Rana hanluica* is an endemic species in China that is largely distributed in the hills and mountains south of the Yangtze River (Shen et al., 2007; Jiang et al., 2021). This species is widely distributed in southern China, with many geographically distinct populations, making it an ideal subject for research. Moreover, its wild populations are facing the risk of being captured and eaten (Xia et al., 2022). Therefore, an in-depth study of the phylogeny of this species and analysis of haplotype spatial distribution can help us understand the migration, dispersal, diversity, and biogeography of amphibians in this region.

Therefore, this study systematically evaluates the phylogeography, spatial distribution of haplotypes, and genetic variation of the nuclear gene RAG2 and mitochondrial gene Cytb in *R. hanluica* specimens collected from 14 distinct mountainous regions (belong to 7 Mountains) in Southern China. China’s topography spans from west to east, featuring highland and lowland landscapes. The terrain includes the Qinghai-Tibet Plateau, reaching an elevation of about 4,000 m; the Terrain II, ranging between 1,000 and 2000 m elevation, is predominant in the western plateau and basin regions; and the Terrain III comprises plains in the eastern region, with elevations lower than 500 m, characterized by scattered hills. The western region generally experiences a moister and warmer climate compared to the eastern part (Yan, et al., 2021).

## Methods

### Sampling

**FIGURE 1:**
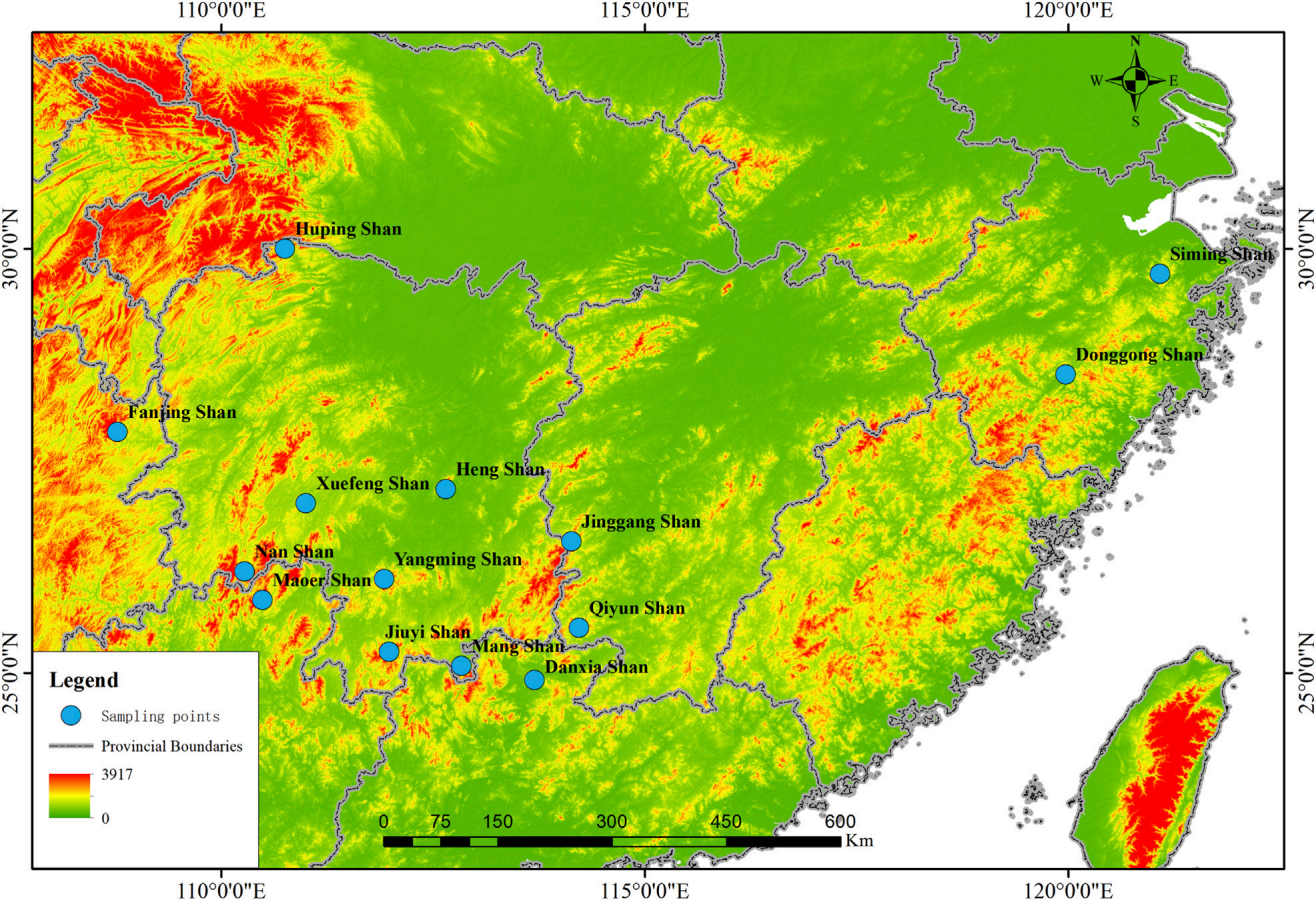
Collection Map Sites: Samples of *Rana hanluica* were collected from 14 mountainous regions in southern China. Each blue badge represents the approximate geographical coordinates of each collection point on the map, with the name of the collection point located adjacent to the blue badge.

Between May 2020 and October 2022, tissue samples from 162 *R. hanluica* individuals were collected across 14 distinct mountainous regions within seven mountain ranges in China (Supplementary Appendix Table S1, Figure 1). For each sample, strict measures were taken to ensure minimal contamination. Individual handling was conducted using new, disposable plastic gloves. Following euthanasia of each specimen in a chloral hydrate solution, liver tissues were promptly collected and flash-frozen at −20°C for subsequent DNA extraction. The remaining tissues underwent a 24-h fixation in formalin before preservation in 75% ethanol (Khatiwada J, et al., 2019).

This comprehensive sampling approach received ethical approval from the Animal Welfare Committee at Central South University of Forestry and Technology (No.20230524118).

### DNA extraction, PCR amplification and sequencing

The genomic DNA extraction was conducted using the Tsingke TSP201-200 DNA extraction kit (https://www.tsingke.net). A partial segment of the mitochondrial gene encoding cytochrome b (Cytb) was successfully amplified from 162 individuals, while partial sequences of the nuclear gene encoding recombination activating gene 2 (RAG2) were amplified from 143 individuals. For the Cytb gene amplification, the primers Cytbs and Cytba were employed, as detailed in the study by Zhou et al. (2012), and L14850 and H15502 primers were utilized following the study by Tanaka et al. (1998). Regarding RAG2, amplification relied on the RAG2s and RAG2a primers outlined in the study by Zhou et al. (2013).

Standard polymerase chain reactions (PCR) were executed in a total volume of 50 μL, employing the following cycling conditions: an initial denaturation step at 98°C for 2 min, followed by 30 cycles of denaturation at 98°C for 10 s, annealing at 55°C for 10 s, extension at 72°C for 10 s, and concluding with a final extension step at 72°C for 5 min. PCR purification and subsequent sequencing processes were carried out by Biomarker Technologies Co. Ltd (China).

### Data analyses

The integrity and high quality of all sequences were assessed through the following methods: 1. Removal of low-resolution extreme values (aberrant DNA sequencing peaks), 2. Observation of dual peaks at individual positions within the sequencing profile, considering only the highest peak, and 3. Multiple sequence alignment of all these sequences using the global ClustalW method in MegaX, followed by the removal of excessive adapter regions from both ends of the sequences, resulting in data matrices suitable for various analyses (Chen et al., 2020). This process yielded Cytb sequences of 616 bp for 162 samples and RAG2 sequences of 426 bp for 143 samples. As of October 2022, all available *R. hanluica* samples of Cytb and RAG2 sequences publicly accessible in GenBank have been incorporated into this study (Kumar et al., 2016).

The Cytb and RAG2 sequences underwent distinct analyses. Utilizing DnaSP 6, we computed several parameters: haplotype count (nh), haplotype diversity (h), nucleotide diversity (π). Roehl network data (*.rdf) files, generated by DnaSP, facilitated subsequent network analysis. NETWORK v.10200 was employed to construct a Median-Joining (MJ) network, unveiling genetic relationships among identified *R. hanluica* haplotypes within and across different regions (Martínez et al., 2019).

Arlequin v3.5 estimated pairwise genetic distances (considered significant when *p* < 0.05), average pairwise differences within and among populations, and conducted an Analysis of Molecular Variance (AMOVA) through 1,000 permutations to assess population genetic structure. Tajima’s D test and Fu’s Fs were computed from 1,000 simulated samples, probing for signatures of selective neutrality or demographic expansion (Ganbold et al., 2020).

The construction of Maximum Likelihood phylogenetic trees for all haplotypes involved a run spanning 10,000 generations within IQtree (v 2.3.2 http://www.iqtree.org/). The selection of the most suitable models or algorithms was guided by recommendations provided by jModelTest. We selected *R. chensinensis*, which is one of the most widely distributed frogs in China, as the outgroup species (Gomez et al., 2012). *R. chensinensis* is believed to have diverged from *R. hanluica* around 13.3 MYA (CI:10.6–15.9 MYA) Timetree (http://timetree.temple.edu/).

## Results

This study delved deeply into Cytb sequences from 162 *R. hanluica* individuals across 14 mountainous regions in southern China. Among these sequences, we discovered 35 single nucleotide polymorphisms (SNPs) loci, neatly dividing the samples into 20 haplotypes (nh = 20). The overall haplotype diversity was *h* = 0.6562 ± 0.0359, with a nucleotide diversity of π = 0.0386 ± 0.0266 (Table 1). Analyzing data from each region, Jiuyi Shan (in the Nanling Mountains) exhibited the highest haplotype diversity (nh = 8, *h* = 0.800). In contrast, Maoer Shan, Fanjing Shan, Nan Shan, and Donggong Shan had the lowest haplotype diversity (nh = 1, *h* = 0.000). These observations aligned with nucleotide diversity, as Jiuyi Shan also had the highest π value (π = 0.1181). When considering the two metrics Tajima’s D and Fu’s Fs, which are used to assess genetic bottlenecks, the Cytb gene of *R. hanlucia* does not exhibit any significant Fu’s Fs values. However, Tajima’s D demonstrates a significant negative value only in the overall population and specifically in the Qiyun Shan subpopulation.

**TABLE 1:** Genetic variability of the Cytb gene in the different sampled localities.

	Number of isolates	Number of haplotypes	Haplotype diversity	Nucleotide diversity	Tajima’s D	Fu’s fs
Heng Shan	5	2	0.4000 ± 0.2373	0.0229 ± 0.0229	−0.9726	1.0404
Jinggang Shan	11	4	0.7091 ± 0.0990	0.0509 ± 0.0389	1.1158	0.5046
Maoer Shan	6	1	0.0000 ± 0.0000	0.0000 ± 0.0001	0.0000	0.0000
Fanjing Shan	9	1	0.0000 ± 0.0000	0.0000 ± 0.0001	0.0000	0.0000
Xuefeng Shan	9	2	0.2000 ± 0.1541	0.0057 ± 0.0087	−1.1117	−0.3393
Yangming Shan	12	2	0.1818 ± 0.1436	0.0052 ± 0.0082	−1.1285	−0.4099
Mang Shan	8	2	0.4286 ± 0.1687	0.0122 ± 0.0139	0.3335	0.5363
Xianxia Shan	11	2	0.1818 ± 0.1436	0.0052 ± 0.0081	−1.1285	−0.4099
Jiuyi Shan	16	8	0.8000 ± 0.0916	0.1180 ± 0.0695	−0.7663	−0.5143
Danxia Shan	25	2	0.4533 ± 0.0717	0.0130 ± 0.0132	1.1805	1.3440
Huping Shan	16	4	0.0130 ± 0.0132	0.0195 ± 0.0175	−0.7079	−1.0977
Nan Shan	14	1	0.0000 ± 0.0000	0.0000 ± 0.0000	0.0000	0.0000
Donggong Shan	6	1	0.0000 ± 0.0000	0.0000 ± 0.0000	0.0000	0.0000
Qiyun Shan	14	4	0.6571 ± 0.0800	0.0533 ± 0.0362	−1.7276^a^	1.1293
ALL	162	20	0.6562 ± 0.0359	0.0386 ± 0.0266	−2.2956^a^	−12.9969

^a^ *p* < 0.05.

**FIGURE 2:**
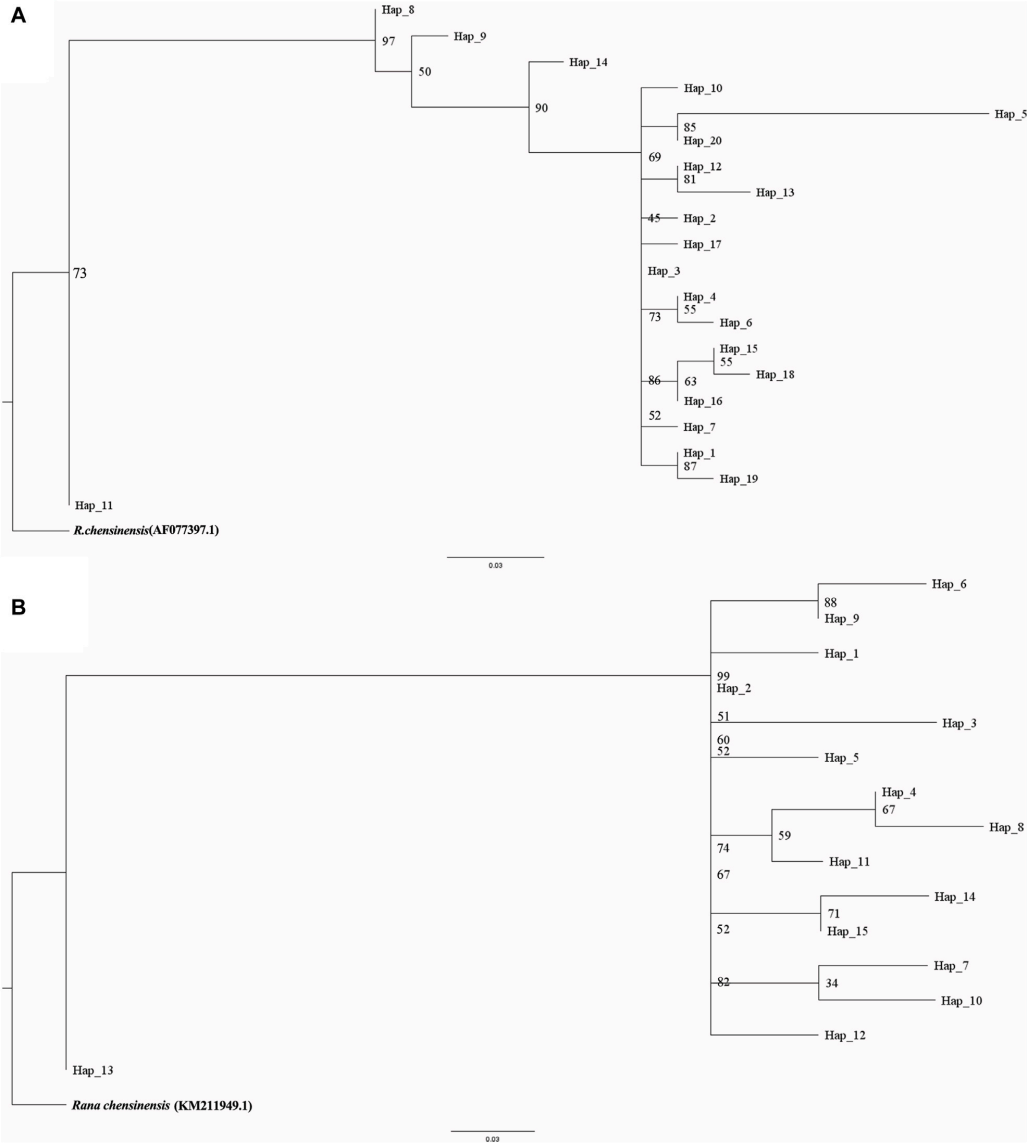
Inferred haplotype phylogeny of *R. hanluica*, utilizing *R. chensinensis* as the outgroup. **(A)** The phylogenetic tree fashioned from the Cytb gene, **(B)** The phylogenetic tree fashioned from the RAG2 gene. The phylogeny was rooted using the genetic common ancestor of both species. Branch lengths, indicated below the tree, represent genetic distance. The numbers along the branches represent bootstrap values.

**FIGURE 3:**
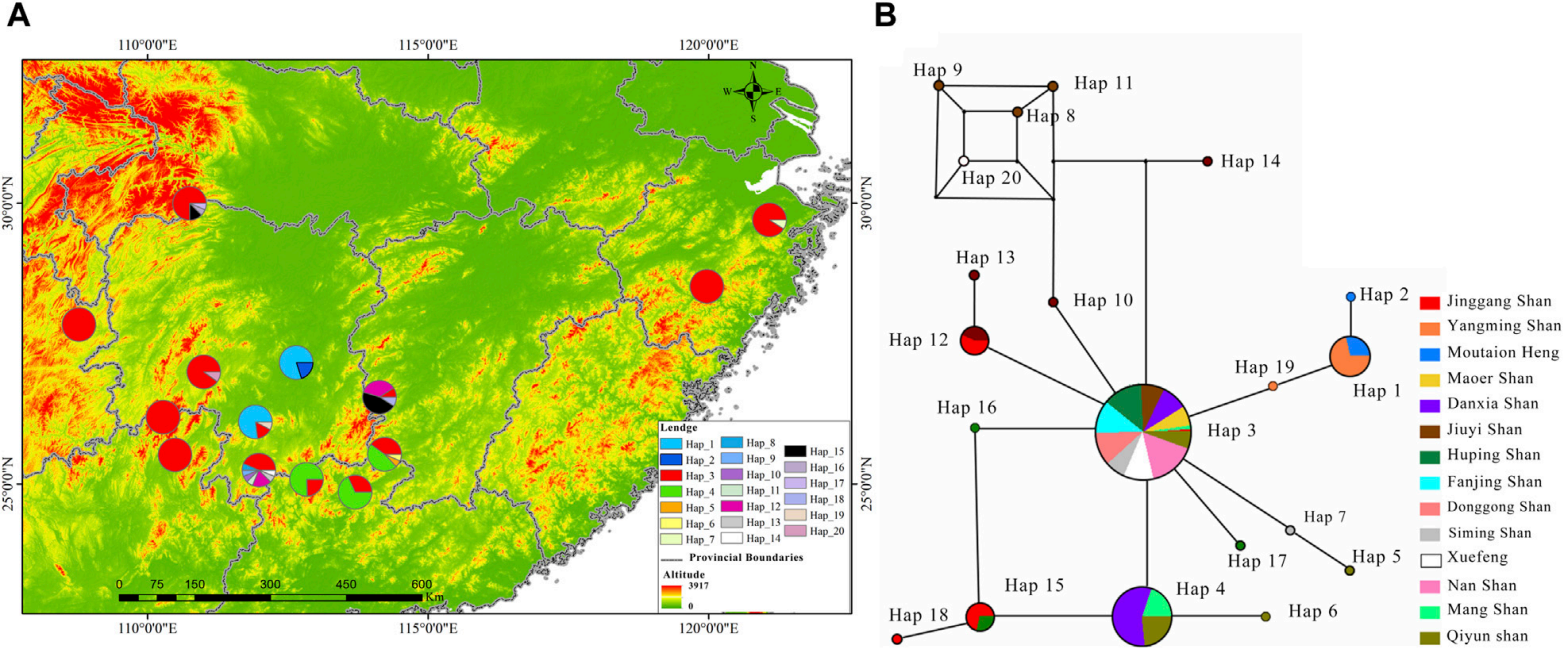
Illustrates the distribution and haplotype network of *R. hanluica*’s Cytb gene in southern China. **(A)** Haplotype distribution: Map of 14 southern Chinese localities showing Cytb haplotype spread. Circles represent different areas, and colors within signify haplotypes 1-20. Color proportions indicate haplotype frequency in each area. **(B)** Haplotype network: Diagram showing lineage haplotypes. Circles represent haplotypes, colors indicate geographical origin, and color ratios reveal the regional proportion of each haplotype.

This rewrite aims to improve readability by using a more narrative style and clearer language, while maintaining the core information. For a comprehensive understanding phylogenetic status of *R. hanluica*, Maximum Likelihood phylogenetic trees were reconstructed for the haplotypes. In the Cytb haplotype phylogenetic tree, Hap 8, Hap 9, Hap 11, and Hap 14 clustered together, while Hap 7, Hap 5, Hap 20, and Hap 10 formed a separate cluster. Similarly, Hap 15, Hap 18, Hap 4, and Hap 6 clustered together, while the remaining haplotypes did not form recognizable sub-branches (Figure 2A). Haplotype analysis of Cytb sequences revealed fascinating insights. Hap 3 emerged as the dominant haplotype, appearing in 89 samples across 12 of the 14 studied populations, absent only in Heng Shan and Yangming Shan (Figure 3A). Within the haplotype network, Hap 3 emerges as the pivotal hub for genetic exchange. Furthermore, Hap 4, Hap 15, and Hap 16 constitute a discernible cycle. An intricate cycle comprising Hap 8, Hap 9, Hap 10, Hap 11, and Hap 20 has been identified, with the majority of these haplotypes originating from Jiuyi Shan. It's worth mentioning that a significant number of these key nodes are situated within the vast Nanling Mountains. Meanwhile, Hap 2, Hap 5, Hap 6, Hap 14 and Hap 17 occupy the outskirts of the haplotype network (Figure 3B). Another Hap 3 marked its presence in 30 samples from three distinct populations. Other haplotypes were less frequent, often confined to one or two populations, with GC content ranging from 48.57% to 68.57%.

Additionally, we calculated Wright’s F-statistic (pairwise Fst) and pairwise differences for 14 *R. hanluica* groups. Cytb sequence pairwise Fst values among these groups ranged from 0.0009 to 0.0057 (average Fst = 0.0023). The least differentiated pair was Heng Shan and Yangming Shan (Fst = 0.0009), while the most differentiated was Heng Shan and Jiuyi Shan. Mean pairwise differences ranged from 0 to 4.1333, with Fanjing Shan showing the highest (Table 2). The maximum PiXY value was between Jinggang Shan and Fanjing Shan (3.7386), and the minimum was between Huping Shan and Nan Shan, and Huping Shan and Xuefeng Shan (0.0909). AMOVA analysis indicated significant within-population genetic differentiation, with 32.14% variation within and 67.86% between populations.

**TABLE 2:** Estimates of Cytb pairwise Fst (below) and average number of pairwise differences between (PiXY, above) and within population (PiX, diagonal) among 14 population.

	S1	S2	S3	S4	S5	S6	S7	S8	S9	S10	S11	S12	S13	S14
S1	0.8000	2.5455	1.0000	1.0000	1.1000	0.5692	2.0000	1.0909	3.3750	1.6800	1.3750	1.0000	1.0000	2.2000
S2	0.0041	1.7818	1.5455	1.5455	1.6455	2.4685	1.4546	1.6364	3.7386	1.4836	1.5796	1.5455	1.5455	2.1636
S3	0.0009	0.0041	0.0000	0.0000	0.1000	0.9231	1.0000	0.0909	2.3750	0.6800	0.3750	0.0000	0.0000	1.2000
S4	0.0033	0.0024	0.0033	0.0000	0.1000	0.9231	1.0000	0.0909	2.3750	0.6800	0.3750	0.0000	0.0000	1.2000
S5	0.0016	0.0025	0.0016	0.0016	0.2000	1.0231	1.1000	0.1909	2.4750	0.7800	0.4750	0.1000	0.1000	1.2867
S6	0.0027	0.0024	0.0027	0.0005	0.0011	0.4359	1.9231	1.0140	3.2981	1.6031	1.2981	0.9231	0.9231	2.1231
S7	0.0055	0.0061	0.0055	0.0055	0.0039	0.0050	0.0000	1.0909	3.3750	0.3200	1.1250	1.0000	1.0000	1.1333
S8	0.0022	0.0026	0.0022	0.0018	0.0006	0.0014	0.0045	0.1818	2.4659	0.7709	0.4659	0.0909	0.0909	1.2909
S9	0.0016	0.0025	0.0016	0.0016	0.0000	0.0011	0.0039	0.0006	4.1333	3.0550	2.7500	2.3750	2.3750	3.5750
S10	0.0018	0.0027	0.0018	0.0018	0.0001	0.0013	0.0040	0.0008	0.0001	0.4533	0.8850	0.6800	0.6800	1.1547
S11	0.0019	0.0027	0.0019	0.0019	0.0002	0.0013	0.0041	0.0008	0.0002	0.0004	0.6833	0.3750	0.3750	1.4417
S12	0.0016	0.0025	0.0016	0.0016	0.0000	0.0011	0.0039	0.0006	0.0000	0.0001	0.0002	0.0000	0.0000	1.2000
S13	0.0016	0.0025	0.0016	0.0016	0.0000	0.0011	0.0039	0.0006	0.0000	0.0001	0.0002	0.0000	0.0000	1.2000
S14	0.0037	0.0036	0.0037	0.0019	0.0021	0.0019	0.0060	0.0025	0.0021	0.0022	0.0023	0.0021	0.0021	1.8667

Heng Shan (S1) Jinggang Shan (S2) Yangming Shan (S3) Mang Shan (S4) Maoer Shan (S5) Danxia Shan (S6) Jiuyi Shan (S7) Huping Shan (S8) Fanjing Shan (S9) Siming Shan (S10) Donggong Shan (S11) Xuefeng Shan (S12) Nan Shan (S13) Qiyun Shan (S14).

We also conducted an in-depth analysis of 143 RAG2 sequences, gathered from the same 14 mountainous regions in southern China as our previous Cytb study. This exploration uncovered 15 SNP loci, resulting in the identification of 15 distinct haplotypes (denoted by nh = 15). The haplotype diversity was measured at *h* = 0.6621 ± 0.0266, accompanied by a nucleotide diversity of π = 0.069 ± 0.051. It’s worth highlighting that Mang Shan, nestled within the Nanling Mountains, exhibited exceptional genetic diversity. With a nh of five and an h of 0.8571, it boasted the highest haplotype diversity among all the sampled locations. Furthermore, its nucleotide diversity, measured at *π* = 0.0976, was also the highest recorded (Table 3). Moreover, for RAG2, Tajima’s D demonstrated a statistically significant negative value (*p* < 0.05) exclusively in Huping Shan, while Fu’s Fs presented a statistically significant negative value in Mang Shan.

**TABLE 3:** Genetic variability of the RAG2 gene in the different sampled localities.

	Number of isolates	Number of haplotypes	Haplotype diversity	Nucleotide diversity	Tajima’s D	Fu’s fs
Heng Shan	5	1	0.0000 ± 0.0000	0.0000 ± 0.0000	0.0000	0.0000
Jinggang Shan	9	5	0.8056 ± 0.1196	0.0889 ± 0.0689	−0.3823	−1.7836
Yangming Shan	12	1	0.0000 ± 0.0000	0.0000 ± 0.0000	0.0000	0.0000
Mang Shan	8	5	0.8571 ± 0.1083	0.0976 ± 0.0753	−0.2218	−1.8588^a^
Maoer Shan	6	1	0.0000 ± 0.0000	0.0009 ± 0.0009	0.0000	0.0000
Danxia Shan	25	5	0.2967 ± 0.1150	0.0209 ± 0.0247	−1.5041	−2.4415^a^
Jiuyi Shan	5	2	0.4000 ± 0.2373	0.0533 ± 0.0533	−0.9726	1.0404
Huping Shan	12	2	0.1538 ± 0.1261	0.0615 ± 0.0509	−1.9297^a^	2.3000
Fanjing Shan	9	2	0.3889 ± 0.1644	0.0778 ± 0.0624	0.2176	2.4087
Xiangxin Shan	11	2	0.3273 ± 0.1533	0.0218 ± 0.0267	−0.1000	0.3563
Donggong Shan	6	1	0.0000 ± 0.0000	0.0000 ± 0.0000	0.0000	0.0000
Xuefeng Shan	7	2	0.5714 ± 0.1195	0.0381 ± 0.0398	1.3416	0.8564
Nan Shan	14	1	0.0000 ± 0.0000	0.0000 ± 0.0000	0.0000	0.0000
Qiyun Shan	14	5	0.7802 ± 0.0846	0.0691 ± 0.0513	−0.3298	−1.3219

^a^ *p* < 0.05.

**FIGURE 4:**
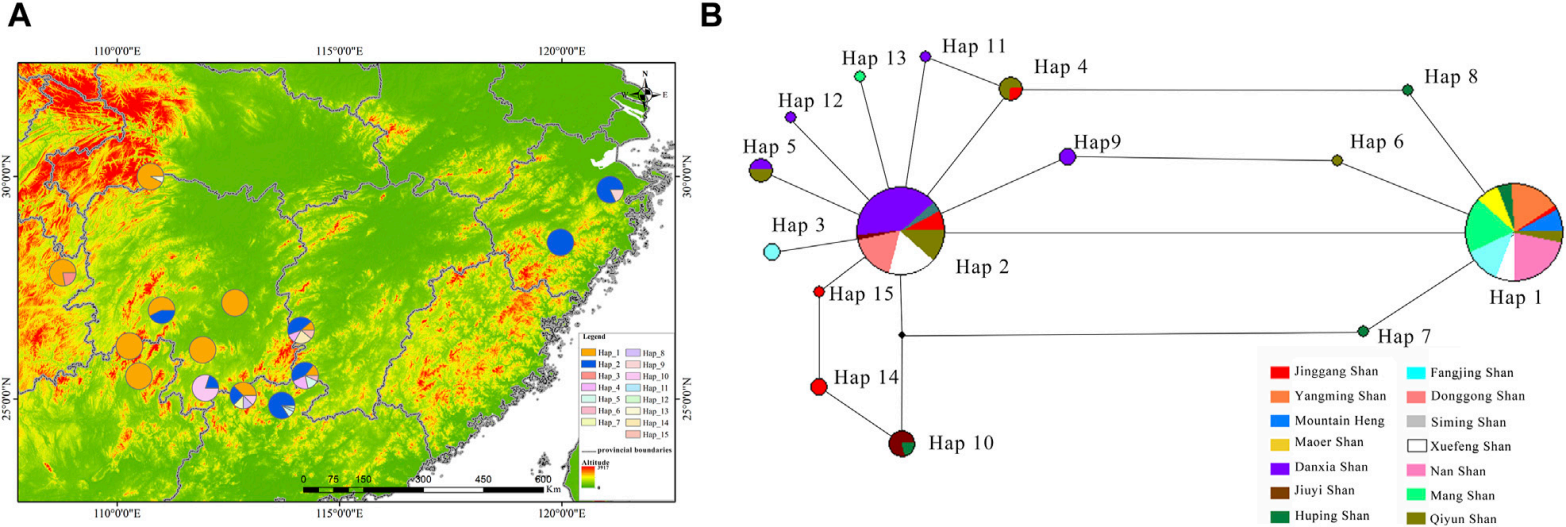
Illustrates the distribution and haplotype network of *R. hanluica*’s RAG2 gene in southern China. **(A)** Haplotype distribution: This map outlines howRAG2 haplotypes are distributed across 14 locations in southern China. Each colored segment within the geographical circles represents haplotypes 1–15, with the size indicating their frequency in that specific area. **(B)** Haplotype network: The diagram uncovers the relationships between various haplotypes. Circles mark different haplotypes, while colors denote their geographical origins. The color proportions within each circle highlight the representation of each region within that haplotype.

The RAG2 haplotype phylogenetic tree indicated Hap13 as the earliest differentiated haplotype (Figure 2A), whereas other haplotypes did not form discernible sub-branches, displaying unique differences among them. Delving deeper into the haplotypes present within the RAG2 gene sequences uncovered fascinating patterns of dispersal. Two different dominant haplotypes are formed in the east and west of Nanling Mountains. The west of Nanling Mountains is dominated by Hap1, while the east is dominated by Hap2 (Figure 4A). In the haplotype network of RAG2, Hap1 and Hap2 stand out as crucial nodes, linking Hap 4, Hap 6, Hap 7, Hap 8, Hap 9, Hap 11, Hap 10, Hap 14, and Hap 15 in multiple cycles. Notably, apart from Hap1 and Hap2, all the intermediate haplotypes mentioned were found exclusively within the Nanling Mountains (Figure 4B). The other haplotypes reside at the outskirts of this haplotype network. Notably, Hap 1 stood out as a dominant haplotype, appearing in 65 samples with a GC content of 66.67%. This haplotype was widespread, being detected in 10 out of the 14 studied populations. Closely following Hap one was Hap 2, which was found in 52 samples, had a GC content of 73.33%, and was present in eight of the 14 populations. In contrast, haplotypes Hap three through Hap 15 was less frequent, often confined to just one or two populations, with GC contents varying between 60.00% and 73.33%.

Regarding genetic differentiation among the 14 populations for RAG2, the pairwise Fst values ranged from 0.0006 to 0.0061, averaging at 0.0028. The least genetically differentiated pair was between Heng Shan and Yangming Shan (Fst = 0.0006), while the most differentiated pair was Heng Shan and Qiyun Shan. The mean pairwise differences within and between populations spanned from 0 to 1.464, with Mang Shan having the highest value (Table 4). The maximum PiXY value was observed in the comparison between Jiuyi Shan and Qiyun Shan (1.555), whereas the minimum was seen between Heng Shan and Yangming Shan, as well as between Heng Shan and Maoer Shan (0.000). Additionally, our AMOVA analysis indicated that about 52.85% of the total variation was due to within-population differences, while 47.15% was attributed to between-population disparities.

**TABLE 4:** Estimates of RAG2 pairwise Fst (below) and average number of pairwise differences between (PiXY, above) and within population (PiX, diagonal) among 14 population.

	S1	S2	S3	S4	S5	S6	S7	S8	S9	S10	S11	S12	S13	S14
S1	0.0000	1.5556	0.0000	0.8753	0.0000	1.1600	2.6000	0.4621	0.6667	1.1817	1.0000	0.4289	0.0000	1.2144
S2	0.0037	1.3333	1.5556	1.6265	1.5556	0.9333	2.0222	1.8799	1.8265	0.9600	0.7778	1.2222	1.5556	1.3246
S3	0.0006	0.0039	0.0000	0.8765	0.0000	1.1600	2.6000	0.4621	0.6667	1.1821	1.0000	0.4291	0.0000	1.2143
S4	0.0012	0.0041	0.0019	1.4643	0.8746	1.2800	2.1253	1.2787	1.3748	1.3067	1.1254	0.9823	0.8753	1.4456
S5	0.0012	0.0041	0.0014	0.0009	0.8746	1.2800	2.1253	0.4621	0.6667	1.1821	1.0000	0.4291	0.0000	1.2143
S6	0.0027	0.0023	0.0031	0.0031	0.0027	1.1600	2.6000	1.4684	1.3821	0.3424	0.1599	0.7312	1.1600	0.7711
S7	0.0057	0.0048	0.0056	0.0064	0.0031	0.0037	1.7600	2.9078	2.8221	1.7822	1.6000	2.1711	2.6000	2.2431
S8	0.0011	0.0041	0.0023	0.0021	0.0011	0.0041	0.0067	0.9231	1.0944	1.4900	1.3081	0.8243	0.4618	1.5545
S9	0.0014	0.0037	0.0024	0.0021	0.0014	0.0037	0.0067	0.0017	1.1667	1.4041	1.2222	0.9045	0.6667	1.5321
S10	0.0028	0.0019	0.0027	0.0031	0.0028	0.0009	0.0041	0.0029	0.0029	0.3273	0.1823	0.7531	1.1817	0.7989
S11	0.0024	0.0019	0.0027	0.0031	0.0024	0.0000	0.0037	0.0029	0.0029	0.0000	0.0000	0.5713	1.0000	0.6433
S12	0.0010	0.0032	0.0009	0.0021	0.0010	0.0017	0.0051	0.0016	0.0020	0.0018	0.0013	0.5713	0.4284	0.9700
S13	0.0009	0.0041	0.0009	0.0009	0.0000	0.0027	0.0061	0.0009	0.0014	0.0028	0.0024	0.0009	0.0000	1.2141
S14	0.0029	0.0032	0.0032	0.0032	0.0029	0.0018	0.0053	0.0041	0.0041	0.0019	0.0009	0.0019	0.0032	1.1321

Heng Shan (S1) Jinggang Shan (S2) Yangming Shan (S3) Mang Shan (S4) Maoer Shan (S5) Danxia Shan (S6) Jiuyi Shan (S7) Huping Shan (S8) Fanjing Shan (S9) Siming Shan (S10) Donggong Shan (S11) Xuefeng Shan (S12) Nan Shan (S13) Qiyun Shan (S14).

## Discussion

Our study represents a significant advancement in the understanding of *R. hanluica*’s genetics by exploring previously unexamined haplotypes, analyzing a larger sample size from the biologically significant Nanling Mountainous, and broadening the geographical scope of genetic diversity research on this species. Across a vast geographical range, both nuclear (RAG2) and mitochondrial (Cytb) genetic data (Yuan et al., 2016), specifically Fst and PiX values, consistently reveal a lack of distinct genetic structuring in *R. hanluica* populations. This finding strongly suggests widespread migratory behavior in this species, encompassing both males and females. Furthermore, the existence of multiple interconnected cycles within the haplotype network indicates significant gene flow among various populations. Phylogenetic analyses, represented by haplotype trees, exhibit extended branch lengths for both Cytb and RAG2, highlighting a remarkable degree of genetic diversity. Based on these comprehensive genetic insights, we posit that *R. hanluica* engages in migratory patterns. Such migrations profoundly influence the species’ distribution and genetic makeup, enabling it to expand its territorial range and enhance its genetic repertoire through inter-population gene exchange. The sampled *R. hanluica* populations showcased remarkable DNA diversity. Our findings highlight several regions, particularly the Nanling Mountains and their intersections with other ranges, exhibiting the highest diversity in both Cytb and RAG2 among the studied populations. Notable locations include Jiuyi Shan (Nanling Mountains), Mang Shan (Nanling Mountains), Jinggang Shan (Belongs to Luoxiao Mountains adjacent to Nanling Mountains), and Qiyun Shan (Belongs to Luoxiao Mountains adjacent to Nanling Mountains). In recent years, numerous cryptic amphibian species have been discovered in southern China, specifically in the Nanling Mountains—a renowned biodiversity hotspot (Luo et al., 2021; Gao et al., 2022; Wang et al., 2022). This remarkable finding can be attributed to the intricate network of east-west valleys that create diverse microclimates in the region. These unique environmental conditions foster species adaptability, potentially driving species radiation (Li et al., 2020). Morphological variations, such as differing limb lengths and head widths, observed in specimens of the Hanlu wood frog (Zhou et al., 2017; Yan et al., 2022) further corroborate the evidence of species radiation within this amphibian group. Additionally, the identified genetic diversity among the Hanlu wood frogs could also stem from this species radiation phenomenon.

Phylogenetic analyses unveiled that the foundational branch predominantly comprised samples from Jiuyi Shan, suggesting a potential origin of *R. hanluica* in the Nanling region, potentially dispersing along the east-west trending mountain ranges. From a haplotype perspective, RAG2 primarily displayed two distinct types, Hap1 and Hap2. Hap1 was primarily distributed in western regions, while Hap2 was more prevalent in eastern areas. Several regions, including Jiuyi Shan, Mang Shan, Jinggang Shan, Qiyun Shan, and Danxia Shan from the Nanling and Luoxiao Mountains, shared these two haplotypes. This distribution pattern suggests that the east-west Nanling Mountains potentially served as a genetic corridor connecting the north-south Wuling Mountains, Xuefeng Mountains, Luoxiao Mountains, and Wuyi Mountains. Multiple studies have also confirmed that the Nanling Mountains in China have a high level of species diversity and harbor a large number of biological species (Li et al., 2015; Tian et al., 2018; Lyu et al., 2020).

Furthermore, the Cytb analysis indicated unique genotypes in Heng Shan and Yangming Shan. This divergence might be attributed to Heng Shan being predominantly surrounded by plains, hindering gene flow with external regions, and resulting in distinct genotypes. However, this does not imply complete isolation between Heng Shan and Yangming Shan. In the haplotype network, Cytb’s Hap1 and Hap2 are connected to Hap3 via Hap19, and in the phylogenetic tree, Hap3 clusters together with Hap1and Hap19. This might suggest that the Heng Shan area is only relatively isolated, and there is gene flow between *R. hanluica* populations in other regions through Yangming Shan (located to the southwest). This research paves the way for a deeper understanding evolutionary journey of *R. hanluica*, highlighting the potential impact of geographical barriers and habitat changes on its genetic diversity and dispersion across Southern China. The haplotype network based on the Cytb gene showed a star-like structure, and some haplotypes were not detected in some places, indicating a recent population expansion. However, the haplotype network based on the RAG2 gene showed a reticulate distribution, with multiple connections between haplotypes from different populations, suggesting high levels of gene flow among *R. hanluica* groups. This may be related to the different rates of evolution of these two genes (Brown et al., 1979). The results of the AMOVA also indicated that genetic variation primarily occurs within populations of *R. hanluica* However, higher genetic differentiation (0.0856–0.7429) reflected the existence of differentiation between populations of *R. hanluica,* possibly due to genetic drift (Zhang et al., 2018). Previous studies have shown that the causes of genetic differentiation in amphibians in southern China are mainly geological history (Tian et al., 2018; Li et al., 2022), climate fluctuations (Li et al., 2018), and sky islands (Shepard and Burbrink, 2009; Pan et al., 2019). The distribution range of *R. hanluica* is in the middle and low altitudes, and its breeding environment is limited by stagnant waters such as ponds and paddy fields. High-altitude areas such as the Nanling and Luoxiao Mountains and large rivers such as the Xiangjiang and Ganjiang may also restrict the migration and diffusion of *R. hanluica* However, the high level of gene flow and single phylogenetic branch both indicate gene exchange between populations of *R. hanluica*. Therefore, the authors believe that, on the one hand, these patterns may be due to the short time since the species differentiated; combined with the influence of genetic drift, the populations have not accumulated enough variation during the evolutionary process. On the other hand, multiple studies have shown that the Nanling Mountains are an important biological corridor, and after the expansion of the *R. hanluica* population, different subpopulations may have migrated and spread through the Nanling Mountains, resulting in secondary contact (Li et al., 2015; Li et al., 2022).

Population bottlenecks typically refer to phenomena where the size of a population experiences a sharp decline due to environmental pressures, disasters, or other factors, resulting in reduced genetic diversity (Weaver et al., 2021). Migration behavior plays a pivotal role in this context. When a group of individuals from a population migrate to a new geographical location seeking a more suitable habitat, they may become isolated from the original population, eventually forming a new, smaller subpopulation. However, due to the limited founding population, the genetic diversity of this new subpopulation may be significantly constrained (Cardenas et al., 2020).

Cytb analysis reveals a statistically significant negative Tajima’s D value, indicating that the species is undergoing a genetic bottleneck, potentially linked to climate change. As Xia et al. (2021) study, key environmental factors influencing the potential geographical distribution of *R. hanlucia* include precipitation during the driest month and altitude. Under future climate change scenarios, suitable habitats for *R. hanlucia* in Hunan and Guizhou provinces are projected to experience significant losses without any compensatory gains. Consequently, suitable habitats for these frogs may shift to higher altitudes, forming isolated ecological niches. While this migratory behavior might facilitate the survival of some individuals, it could also contribute to a reduction in population size and genetic diversity (Pauls et al., 2013).

Moreover, the correlation between population loss and population bottlenecks cannot be overlooked. In specific regions of Jiangxi, Hunan, and Zhejiang, human predation poses a threat to this species, potentially leading to a rapid decline in its population (Xia et al., 2022). Once this loss crosses a threshold, it can precipitate a population bottleneck. Inevitably, during such bottlenecks, genetic diversity diminishes, posing a threat to the population’s adaptability and long-term survival. The notable negative Tajima’s D value observed in Cytb samples from Qiyun Shan may be indicative of this trend.

Additionally, the prominent negative Fu’s Fs value for RAG2 in Mang Shan hints at a possible population recovery after undergoing a genetic bottleneck. This recovery might be attributed to the intensified conservation measures implemented in the Hunan Mangshan National Nature Reserve since 2016. These efforts, primarily aimed at safeguarding the Mangshan pit viper, as stated by the National Forestry and Grassland Administration of China (https://www.forestry.gov.cn/), could have contributed to the observed population resurgence.

## Conclusion

The study dives into genetic intricacies of *R. hanluica* in Southern China, using Cytb and RAG2 regions to probe its population structures, diversity, and origins. These genetic markers, known for decoding evolutionary histories, offer a window into complex journey of *R. hanluica*. The sampled populations painted a diverse genetic picture, notably in Nanling Mountains regions like Jiuyi Shan, Jinggang Shan, Mang Shan, and Qiyun Shan, revealing hubs of genetic richness. Yet, further exploration is needed to validate these findings and decipher precise origins of *R. hanluica*.

Phylogenetic analyses hint at probable origin of *R. hanluica* in the Nanling region, dispersing along east-west mountain ranges, notably from Jiuyi Shan samples. RAG2 gene patterns disclosed unique haplotype distributions between west and east, suggesting historical migratory routes and barriers affecting the species’ evolution. Distinct genetic profiles in Heng Shan and Yangming Shan propose geographic isolation, shaping unique genotypes and raising questions about geographical barriers’ role in genetic diversity and dispersal in Southern China.

These insights hold critical implications for conservation efforts. Identifying high-diversity regions and potential gene flow corridors is crucial for effective conservation strategies. Preserving genetic integrity, especially in regions like the Nanling Mountains, promises to safeguard evolutionary potential of *R. hanluica*. This study lays the groundwork for understanding genetic landscape of *R. hanluica,* illuminating its origins, population structures, and evolutionary pathways, offering guidance for future research and conservation endeavors.

## Data Availability

The datasets presented in this study can be found in online repositories. The names of the repository/repositories and accession number(s) can be found in the article/Supplementary Material.
