# Supplementary material for: Genetic diversity and haplotype distribution patterns analysis of cytb and RAG2 sequences in Rana hanluica from southern China
Source: Front Genet. 2024 May 20;15:1374263. doi: 10.3389/fgene.2024.1374263 (PMC11145506; doi:10.3389/fgene.2024.1374263)
Supplement: Supplementary file 1 [file Table1.pdf]

Appendix Table. Detailed information for samples.

**Voucher No.**	**location**	**mountain range**	**Cytb** Hap	**RAG2 Hap**	**Cytb**	**RAG2**
CN01	Heng Shan	Mountain Heng	C-H1	R-H1	OQ982954	OQ983116
CN02	Heng Shan	Mountain Heng	C-H1	R-H1	OQ982955	OQ983117
CN03	Heng Shan	Mountain Heng	C-H1	R-H1	OQ982956	OQ983118
CN04	Heng Shan	Mountain Heng	C-H2	R-H1	OQ982957	OQ983119
CN05	Heng Shan	Mountain Heng	C-H1	R-H1	OQ982958	OQ983120
GD06	Qiyun Shan	Luoxiao Mountains	C-H3	R-H2	OQ982959	OQ983121
GX01	Maoer Shan	Nanling Mountains	C-H3	—	OQ982960	—
GX02	Maoer Shan	Nanling Mountains	C-H3	R-H1	OQ982961	OQ983122
GX03	Maoer Shan	Nanling Mountains	C-H3	R-H1	OQ982962	OQ983123
GX04	Maoer Shan	Nanling Mountains	C-H3	R-H1	OQ982963	OQ983124
GX05	Maoer Shan	Nanling Mountains	C-H3	R-H1	OQ982964	OQ983125
GX06	Maoer Shan	Nanling Mountains	C-H3	R-H1	OQ982965	OQ983126
GZ02	Fanjing Shan	Wuling Mountains	C-H3	R-H1	OQ982966	OQ983127
GZ03	Fanjing Shan	Wuling Mountains	C-H3	R-H1	OQ982967	OQ983128
GZ04	Fanjing Shan	Wuling Mountains	C-H3	R-H1	OQ982968	OQ983129
GZ05	Fanjing Shan	Wuling Mountains	C-H3	R-H1	OQ982969	OQ983130
GZ06	Fanjing Shan	Wuling Mountains	C-H3	R-H2	OQ982970	OQ983131
GZ07	Fanjing Shan	Wuling Mountains	C-H3	R-H2	OQ982971	OQ983132
GZ16	Fanjing Shan	Wuling Mountains	C-H3	R-H1	OQ982972	OQ983133
GZ17	Fanjing Shan	Wuling Mountains	C-H3	R-H1	OQ982973	OQ983134
HJ01	Xuefeng Shan	Xuefeng Mountains	C-H3	R-H1	OQ982974	OQ983135
HJ02	Xuefeng Shan	Xuefeng Mountains	C-H3	—	OQ982975	—
HJ03	Xuefeng Shan	Xuefeng Mountains	C-H3	—	OQ982976	—
HJ04	Xuefeng Shan	Xuefeng Mountains	C-H3	R-H1	OQ982977	OQ983136
HJ05	Xuefeng Shan	Xuefeng Mountains	C-H3	R-H1	OQ982978	OQ983137
HJ06	Xuefeng Shan	Xuefeng Mountains	C-H3	R-H1	OQ982979	OQ983138
HJ07	Xuefeng Shan	Xuefeng Mountains	C-H3	R-H2	OQ982980	OQ983139
JX01	Qiyun Shan	Luoxiao Mountains	C-H3	R-H4	OQ982981	OQ983140
JX03	Qiyun Shan	Luoxiao Mountains	C-H4	R-H2	OQ982982	OQ983141
JX04	Qiyun Shan	Luoxiao Mountains	C-H4	R-H4	OQ982983	OQ983142
JX05	Qiyun Shan	Luoxiao Mountains	C-H4	R-H5	OQ982984	OQ983143
JX06	Qiyun Shan	Luoxiao Mountains	C-H5	R-H4	OQ982985	OQ983144
JX07	Qiyun Shan	Luoxiao Mountains	C-H4	R-H2	OQ982986	OQ983145
JX08	Qiyun Shan	Luoxiao Mountains	C-H4	R-H5	OQ982987	OQ983146
JX09	Qiyun Shan	Luoxiao Mountains	C-H6	R-H2	OQ982988	OQ983147
JX10	Qiyun Shan	Luoxiao Mountains	C-H4	R-H2	OQ982989	OQ983148
JX11	Qiyun Shan	Luoxiao Mountains	C-H4	R-H6	OQ982990	OQ983149
JX12	Qiyun Shan	Luoxiao Mountains	C-H3	R-H2	OQ982991	OQ983150
JX13	Qiyun Shan	Luoxiao Mountains	C-H3	R-H1	OQ982992	OQ983151
JX14	Qiyun Shan	Luoxiao Mountains	C-H3	R-H1	OQ982993	OQ983152
MS01	Mang Shan	Nanling Mountains	C-H4	R-H1	OQ982994	OQ983153
MS02	Mang Shan	Nanling Mountains	C-H4	R-H2	OQ982995	OQ983154

MS03	Mang Shan	Nanling Mountains	C-H4	R-H7	OQ982996	OQ983155
MS05	Mang Shan	Nanling Mountains	C-H4	R-H1	OQ982997	OQ983156
MS06	Mang Shan	Nanling Mountains	C-H4	R-H1	OQ982998	OQ983157
MS08	Mang Shan	Nanling Mountains	C-H4	R-H8	OQ982999	OQ983158
NB001	Siming Shan	Wuyi Mountains	C-H3	R-H2	OQ983000	OQ983159
NB002	Siming Shan	Wuyi Mountains	C-H3	R-H2	OQ983001	OQ983160
NB003	Siming Shan	Wuyi Mountains	C-H3	R-H2	OQ983002	OQ983161
NB004	Siming Shan	Wuyi Mountains	C-H3	R-H2	OQ983003	OQ983162
NB005	Siming Shan	Wuyi Mountains	C-H3	R-H2	OQ983004	OQ983163
NB006	Siming Shan	Wuyi Mountains	C-H3	R-H9	OQ983005	OQ983164
NB007	Siming Shan	Wuyi Mountains	C-H3	R-H2	OQ983006	OQ983165
NB008	Siming Shan	Wuyi Mountains	C-H7	R-H2	OQ983007	OQ983166
NB009	Siming Shan	Wuyi Mountains	C-H3	R-H9	OQ983008	OQ983167
NB010	Siming Shan	Wuyi Mountains	C-H3	R-H2	OQ983009	OQ983168
NY01	Jiuyi Shan	Nanling Mountains	C-H3	R-H2	OQ983010	OQ983169
NY02	Jiuyi Shan	Nanling Mountains	C-H3	R-H10	OQ983011	OQ983170
NY03	Jiuyi Shan	Nanling Mountains	C-H3	R-H10	OQ983012	OQ983171
NY04	Jiuyi Shan	Nanling Mountains	C-H3	R-H10	OQ983013	OQ983172
NY05	Jiuyi Shan	Nanling Mountains	C-H3	R-H10	OQ983014	OQ983173
NY06	Jiuyi Shan	Nanling Mountains	C-H8	—	OQ983015	—
NY07	Jiuyi Shan	Nanling Mountains	C-H9	—	OQ983016	—
NY08	Jiuyi Shan	Nanling Mountains	C-H10	—	OQ983017	—
NY09	Jiuyi Shan	Nanling Mountains	C-H3	—	OQ983018	—
NY10	Jiuyi Shan	Nanling Mountains	C-H11	—	OQ983019	—
NY11	Jiuyi Shan	Nanling Mountains	C-H12	—	OQ983020	—
NY12	Jiuyi Shan	Nanling Mountains	C-H13	—	OQ983021	—
NY13	Jiuyi Shan	Nanling Mountains	C-H14	—	OQ983022	—
NY14	Jiuyi Shan	Nanling Mountains	C-H3	—	OQ983023	—
NY15	Jiuyi Shan	Nanling Mountains	C-H12	—	OQ983024	—
NY16	Jiuyi Shan	Nanling Mountains	C-H12	—	OQ983025	—
RH01	Danxia Shan	Nanling Mountains	C-H4	R-H2	OQ983026	OQ983174
RH02	Danxia Shan	Nanling Mountains	C-H4	R-H2	OQ983027	OQ983175
RH03	Danxia Shan	Nanling Mountains	C-H4	R-H2	OQ983028	OQ983176
RH04	Danxia Shan	Nanling Mountains	C-H4	R-H2	OQ983029	OQ983177
RH05	Danxia Shan	Nanling Mountains	C-H3	R-H2	OQ983030	OQ983178
RH06	Danxia Shan	Nanling Mountains	C-H3	R-H2	OQ983031	OQ983179
RH07	Danxia Shan	Nanling Mountains	C-H4	R-H2	OQ983032	OQ983180
RH08	Danxia Shan	Nanling Mountains	C-H4	R-H2	OQ983033	OQ983181
RH09	Danxia Shan	Nanling Mountains	C-H4	R-H2	OQ983034	OQ983182
RH10	Danxia Shan	Nanling Mountains	C-H4	R-H2	OQ983035	OQ983183
RH11	Danxia Shan	Nanling Mountains	C-H3	R-H2	OQ983036	OQ983184
RH12	Danxia Shan	Nanling Mountains	C-H3	R-H2	OQ983037	OQ983185
RH13	Danxia Shan	Nanling Mountains	C-H3	R-H2	OQ983038	OQ983186
RH14	Danxia Shan	Nanling Mountains	C-H4	R-H2	OQ983039	OQ983187

RH15	Danxia Shan	Nanling Mountains	C-H4	R-H2	OQ983040	OQ983188
RH16	Danxia Shan	Nanling Mountains	C-H4	R-H2	OQ983041	OQ983189
RH17	Danxia Shan	Nanling Mountains	C-H4	R-H2	OQ983042	OQ983190
RH18	Danxia Shan	Nanling Mountains	C-H4	R-H11	OQ983043	OQ983191
RH19	Danxia Shan	Nanling Mountains	C-H3	R-H2	OQ983044	OQ983192
RH20	Danxia Shan	Nanling Mountains	C-H4	R-H12	OQ983045	OQ983193
RH21	Danxia Shan	Nanling Mountains	C-H3	R-H5	OQ983046	OQ983194
RH22	Danxia Shan	Nanling Mountains	C-H4	R-H5	OQ983047	OQ983195
RH23	Danxia Shan	Nanling Mountains	C-H4	R-H2	OQ983048	OQ983196
RH24	Danxia Shan	Nanling Mountains	C-H3	R-H2	OQ983049	OQ983197
RH25	Danxia Shan	Nanling Mountains	C-H4	R-H2	OQ983050	OQ983198
RZ001	Siming Shan	Wuyi Mountains	C-H3	R-H2	OQ983051	OQ983199
SM02	Huping Shan	Wuling Mountains	C-H3	R-H1	OQ983052	OQ983200
SM03	Huping Shan	Wuling Mountains	C-H3	R-H1	OQ983053	OQ983201
SM04	Huping Shan	Wuling Mountains	C-H3	R-H1	OQ983054	OQ983202
SM05	Huping Shan	Wuling Mountains	C-H3	R-H1	OQ983055	OQ983203
SM06	Huping Shan	Wuling Mountains	C-H3	R-H1	OQ983056	OQ983204
SM07	Huping Shan	Wuling Mountains	C-H3	R-H1	OQ983057	OQ983205
SM08	Huping Shan	Wuling Mountains	C-H3	R-H1	OQ983058	OQ983206
SN01	Nan Shan	Nanling Mountains	C-H3	R-H1	OQ983059	OQ983207
SN02	Nan Shan	Nanling Mountains	C-H3	R-H1	OQ983060	OQ983208
SN03	Nan Shan	Nanling Mountains	C-H3	R-H1	OQ983061	OQ983209
SN04	Nan Shan	Nanling Mountains	C-H3	R-H1	OQ983062	OQ983210
SN05	Nan Shan	Nanling Mountains	C-H3	R-H1	OQ983063	OQ983211
SN06	Nan Shan	Nanling Mountains	C-H3	R-H1	OQ983064	OQ983212
SP01	Yangming Shan	Nanling Mountains	C-H1	R-H1	OQ983065	OQ983213
SP02	Yangming Shan	Nanling Mountains	C-H3	R-H10	OQ983066	OQ983214
SYSa002233	Fanjing Shan	Wuling Mountains	C-H3	R-H1	OQ983068	OQ983216
SYSa004346	Fanjing Shan	Wuling Mountains	C-H3	R-H1	OQ983067	OQ983215
SZ01	Huping Shan	Wuling Mountains	C-H15	—	OQ983069	—
SZ02	Huping Shan	Wuling Mountains	C-H15	—	OQ983070	—
SZ03	Huping Shan	Wuling Mountains	C-H3	R-H2	OQ983071	OQ983217
SZ04	Huping Shan	Wuling Mountains	C-H16	R-H13	OQ983072	OQ983218
SZ05	Huping Shan	Wuling Mountains	C-H3	—	OQ983073	—
SZ06	Huping Shan	Wuling Mountains	C-H17	R-H2	OQ983074	OQ983219
SZ07	Huping Shan	Wuling Mountains	C-H3	R-H2	OQ983075	OQ983220
SZ08	Huping Shan	Wuling Mountains	C-H3	R-H2	OQ983076	OQ983221
SZ09	Huping Shan	Wuling Mountains	C-H3	R-H2	OQ983077	OQ983222
TD01	Nan Shan	Nanling Mountains	C-H3	R-H1	OQ983078	OQ983223
TD02	Nan Shan	Nanling Mountains	C-H3	R-H1	OQ983079	OQ983224
TD03	Nan Shan	Nanling Mountains	C-H3	R-H1	OQ983080	OQ983225
TD04	Nan Shan	Nanling Mountains	C-H3	R-H1	OQ983081	OQ983226
TD05	Nan Shan	Nanling Mountains	C-H3	R-H1	OQ983082	OQ983227
TD06	Nan Shan	Nanling Mountains	C-H3	R-H1	OQ983083	OQ983228

TD08	Nan Shan	Nanling Mountains	C-H3	R-H1	OQ983084	OQ983229
TD09	Nan Shan	Nanling Mountains	C-H3	R-H1	OQ983085	OQ983230
XH01	Xuefeng Shan	Xuefeng Mountains	C-H3	R-H2	OQ983086	OQ983231
XH02	Xuefeng Shan	Xuefeng Mountains	C-H3	R-H2	OQ983087	OQ983232
YL01	Jinggang Shan	Luoxiao Mountains	C-H15	R-H14	OQ983088	OQ983233
YL03	Jinggang Shan	Luoxiao Mountains	C-H15	R-H2	OQ983089	OQ983234
YL04	Jinggang Shan	Luoxiao Mountains	C-H15	R-H15	OQ983090	OQ983235
YL05	Jinggang Shan	Luoxiao Mountains	C-H18	R-H1	OQ983091	OQ983236
YL06	Jinggang Shan	Luoxiao Mountains	C-H15	R-H4	OQ983092	OQ983237
YL08	Jinggang Shan	Luoxiao Mountains	C-H12	—	OQ983093	—
YL09	Jinggang Shan	Luoxiao Mountains	C-H12	—	OQ983094	—
YL10	Jinggang Shan	Luoxiao Mountains	C-H12	R-H14	OQ983095	OQ983238
YL11	Jinggang Shan	Luoxiao Mountains	C-H12	R-H2	OQ983096	OQ983239
YL12	Jinggang Shan	Luoxiao Mountains	C-H3	R-H2	OQ983097	OQ983240
YL13	Jinggang Shan	Luoxiao Mountains	C-H14	R-H2	OQ983098	OQ983241
YMS01	Yangming Shan	Nanling Mountains	C-H1	R-H1	OQ983099	OQ983242
YMS02	Yangming Shan	Nanling Mountains	C-H1	R-H1	OQ983100	OQ983243
YMS03	Yangming Shan	Nanling Mountains	C-H1	R-H1	OQ983101	OQ983244
YMS04	Yangming Shan	Nanling Mountains	C-H19	R-H1	OQ983102	OQ983245
YMS05	Yangming Shan	Nanling Mountains	C-H1	R-H1	OQ983103	OQ983246
YMS06	Yangming Shan	Nanling Mountains	C-H1	R-H1	OQ983104	OQ983247
YMS07	Yangming Shan	Nanling Mountains	C-H1	R-H1	OQ983105	OQ983248
YMS08	Yangming Shan	Nanling Mountains	C-H1	R-H1	OQ983106	OQ983249
YMS09	Yangming Shan	Nanling Mountains	C-H1	R-H1	OQ983107	OQ983250
YMS10	Yangming Shan	Nanling Mountains	C-H1	R-H1	OQ983108	OQ983251
ZJ01	Donggong Shan	Wuyi Mountains	C-H3	R-H2	OQ983109	OQ983252
ZJ02	Donggong Shan	Wuyi Mountains	C-H3	R-H2	OQ983110	OQ983253
ZJ03	Donggong Shan	Wuyi Mountains	C-H3	R-H2	OQ983111	OQ983254
ZJ04	Donggong Shan	Wuyi Mountains	C-H3	R-H2	OQ983112	OQ983255
ZJ05	Donggong Shan	Wuyi Mountains	C-H3	R-H2	OQ983113	OQ983256
ZJKD01	Donggong Shan	Wuyi Mountains	C-H3	R-H2	OQ983114	OQ983257
ZJKD02	Donggong Shan	Wuyi Mountains	C-H20	R-H2	OQ983115	OQ983258

."
